# A deep learning model for real-time recognition of immature persimmons in complex field scenarios

**DOI:** 10.3389/fpls.2026.1785493

**Published:** 2026-03-20

**Authors:** Guoqing Chen, Jinfeng Wang, Andrey Kalinin, Jinyan Ju, Ilia Sarnetskii, Rui Xu, Minyi Zhao, Chunxiang Yang

**Affiliations:** 1 College of Engineering, Northeast Agricultural University, Harbin, China; 2 Engineering and Technology Institute, Sankt-Peterburgskij Gosudarstvennyj Agrarnyj Universitet, Saint Petersburg, Russia; 3 Mechanical Engineering College, Heilongjiang University of Science and Technology, Harbin, China

**Keywords:** convolutional neural network, deep learning, persimmon, real-time recognition, YOLOv12

## Abstract

**Introduction:**

Immature persimmons in natural field environments exhibit characteristics such as weak color distinctiveness, small target size, and frequent occlusion by branches and leaves, as well as interference caused by illumination variations. These factors significantly reduce the accuracy of real time detection.

**Methods:**

To address this issue, this study proposes a real-time detection method for immature persimmons based on an improved YOLOv12n framework. Specifically, an image dataset of immature persimmon fruits was constructed under complex real-field conditions, encompassing diverse lighting conditions, occlusion levels, and shooting viewpoints. Multiple data augmentation strategies were employed to enhance sample diversity and improve the generalization capability of the model. Furthermore, MobileViTv3, CBAM, and DySample modules were integrated into the YOLOv12n architecture to simultaneously improve detection accuracy and inference speed. The effectiveness of MCD-YOLOv12n was comprehensively validated through Grad-CAM visualization, ablation studies, and comparative experiments, and its performance was evaluated against various classical models across different real-world scenarios.

**Results:**

Experimental results demonstrate that MCD-YOLOv12n achieves a Precision of 96.30%, a Recall of 89.90%, and an mAP of 95.30% on the persimmon fruit dataset, while maintaining a real-time inference speed of 72.5FPS. Moreover, the proposed model significantly outperforms mainstream object detection approaches, including DETR, EfficientDet, Faster R-CNN, YOLOv10n, and YOLOv12n, thereby satisfying the practical requirements of field operations.

**Discussion:**

These findings contribute to the advancement of smart agriculture and indicate strong application potential for real-time immature persimmon fruit detection and early-stage precision field management.

## 1 Introduction

Persimmon is an economically and nutritionally valuable cash crop, and both fruit yield and quality play a critical role in farmers’ income and regional economic stability (Jia et al., 2024). However, management practices in the persimmon industry have long focused predominantly on the fruit maturation and harvesting stages, often overlooking the early phases of fruit growth and development. Throughout the persimmon growth cycle, the immature stage represents a pivotal period characterized by fruit initiation, differentiation, and enlargement. During this stage, the spatial distribution and growth status of fruits directly reflect the physiological condition of the trees and their adaptability to environmental factors, thereby largely determining the final yield and fruit quality (Han et al., 2024). Compared with the mature stage, the immature stage enables earlier acquisition of fruit-related information. This early insight provides forward-looking data support for dynamic analysis of fruit expansion, regulation of nutrient allocation, and prediction of yield trends. Moreover, accurate identification of immature persimmon fruits is essential for early-stage precision orchard management. In practical production, management operations such as fruit thinning, pruning, and nutrient regulation are typically implemented during the immature stage, and the effectiveness of these decisions directly influences fruit size, uniformity, and overall yield (Ul Hasan et al., 2025). Nevertheless, due to the lack of precise perception of fruit quantity and spatial distribution, management decisions often rely on manual sampling or empirical judgment, which are labor-intensive, inefficient, and highly subjective. Such approaches fail to meet the requirements of refined, large-scale, and intelligent precision agriculture. Therefore, there is an urgent need to develop a method capable of real-time identification of immature persimmon fruits.

With the rapid advancement of modern information technologies, remote sensing, sensor systems, and artificial intelligence have been widely applied in the agricultural domain (Benfenati et al., 2025; Ju et al., 2024). At present, technologies for precision crop monitoring mainly include satellite remote sensing (Hussain et al., 2025), hyperspectral imaging (Wang et al., 2023), LiDAR (Pak and Son, 2025), and machine vision (Wang et al., 2025c). Satellite remote sensing is characterized by broad spatial coverage and strong time-series observation capabilities, and it has demonstrated clear advantages in large-scale field area estimation, crop growth monitoring, and regional early warning of pests and diseases. However, satellite-based observations are highly susceptible to cloud cover and weather conditions, and their spatial resolution is generally insufficient to meet the requirements of single-fruit-level precise identification (Muturi et al., 2025). In contrast, hyperspectral imaging can acquire detailed spectral information of persimmon fruits across continuous narrow wavelength bands, enabling effective characterization of internal compositional differences and physiological states. Nevertheless, its high data acquisition and processing costs, along with relatively slow imaging speed, substantially limit its applicability in real-time persimmon fruit detection and practical field scenarios (Bourriz et al., 2025). LiDAR technology exhibits outstanding performance in three-dimensional structural reconstruction and canopy parameter extraction. However, persimmon fruits are mainly distributed within the plant canopy and are subject to severe occlusion. Moreover, the high cost of LiDAR equipment and the complexity of system integration make it difficult to satisfy the requirements of precise fruit identification and localization (Longani and Kim, 2025). In contrast, machine vision offers notable advantages, including low hardware cost, convenient data acquisition, and high computational efficiency, enabling intuitive perception of persimmon fruits in complex orchard environments (Kuan et al., 2025). Consequently, conducting research on immature persimmon recognition based on machine vision represents a critical technological pathway toward achieving early-stage precise perception and intelligent management of persimmon orchards.

With the rapid advancement of deep learning technologies, object detection methods based on convolutional neural networks (CNNs) have made significant progress in the field of fruit recognition. For instance, Wang et al. (2024a) strengthened the correlation between different channels in the YOLOv8 model by introducing the attention mechanism, and improved the recognition ability of the model to strawberry fruits under complex illumination and occlusion conditions. This work provides a practical reference for harvesting robot applications. Wang et al. (2025a) constructed a large-scale image dataset covering multiple maturity stages and diverse interference conditions. Based on this dataset, they developed a YOLOv9c-based object detection model. The proposed model enabled accurate detection and classification of bananas at different maturity stages. Wang et al. (2025d) proposed a lightweight improved model for real scenes based on the YOLOv11 framework to solve the problems of citrus fruits being easily occluded, small target scale and serious degradation of marginalized features in complex orchard environment. By significantly compressing the number of model parameters without compromising real-time performance, the proposed approach achieves notable improvements in target detection accuracy and overall robustness. However, the above studies mainly focus on the mature stage with obvious fruit color and prominent target features, and the recognition ability of immature fruits under weak saliency and small-scale conditions is still insufficient. In real field environments, immature persimmon fruits exhibit strong similarities to surrounding leaves and branches in both color and texture (Gao et al., 2025). Consequently, conventional detection models are highly susceptible to background interference, leading to frequent missed detections and false positives. This poses greater challenges to the feature representation and multi-scale perception capabilities of detection algorithms. Notably, CBAM (Ma et al., 2023) adaptively reweights feature responses along both channel and spatial dimensions. This design enables the model to focus more effectively on key regions and feature channels associated with immature persimmons. As a result, interference caused by complex backgrounds and varying illumination conditions is significantly suppressed. In addition, MobileViTBv3 and DySample can dynamically regulate the feature reconstruction process while preserving model lightweight design and real-time inference capability. These modules effectively compensate for the limitations of traditional convolutional networks in modeling long-range dependencies (Wang et al., 2025b). As a result, the model achieves a stronger understanding of global fruit structural characteristics and contextual information. Nevertheless, to the best of our knowledge, there have been no reports on the application of these techniques to real-time persimmon fruit detection.

Based on the above background, this study addresses the challenges associated with immature persimmon fruit recognition in natural environments, including low visual saliency, small object size, and complex occlusion. An immature persimmon detection approach based on an improved YOLOv12 framework is therefore proposed. Driven by the requirements of fruit growth monitoring and precision management, this work develops a fruit detection method that achieves a favorable balance between recognition accuracy and real-time performance. The main innovations and contributions of this study are summarized as follows: (1) a persimmon fruit image dataset was established, covering diverse illumination conditions, shooting angles, and levels of background complexity; (2) a real-time persimmon fruit recognition CNN was constructed based on the YOLOv12n architecture; (3) the superiority and effectiveness of the proposed model were systematically validated through ablation studies and comparative experiments; and (4) MCD-YOLOv12n was evaluated in multiple real-world scenarios to qualitatively analyze its discrimination capability and practical application potential.

## 2 Materials and methods

### 2.1 Data acquisition

#### 2.1.1 Image acquisition

**Figure 1:**
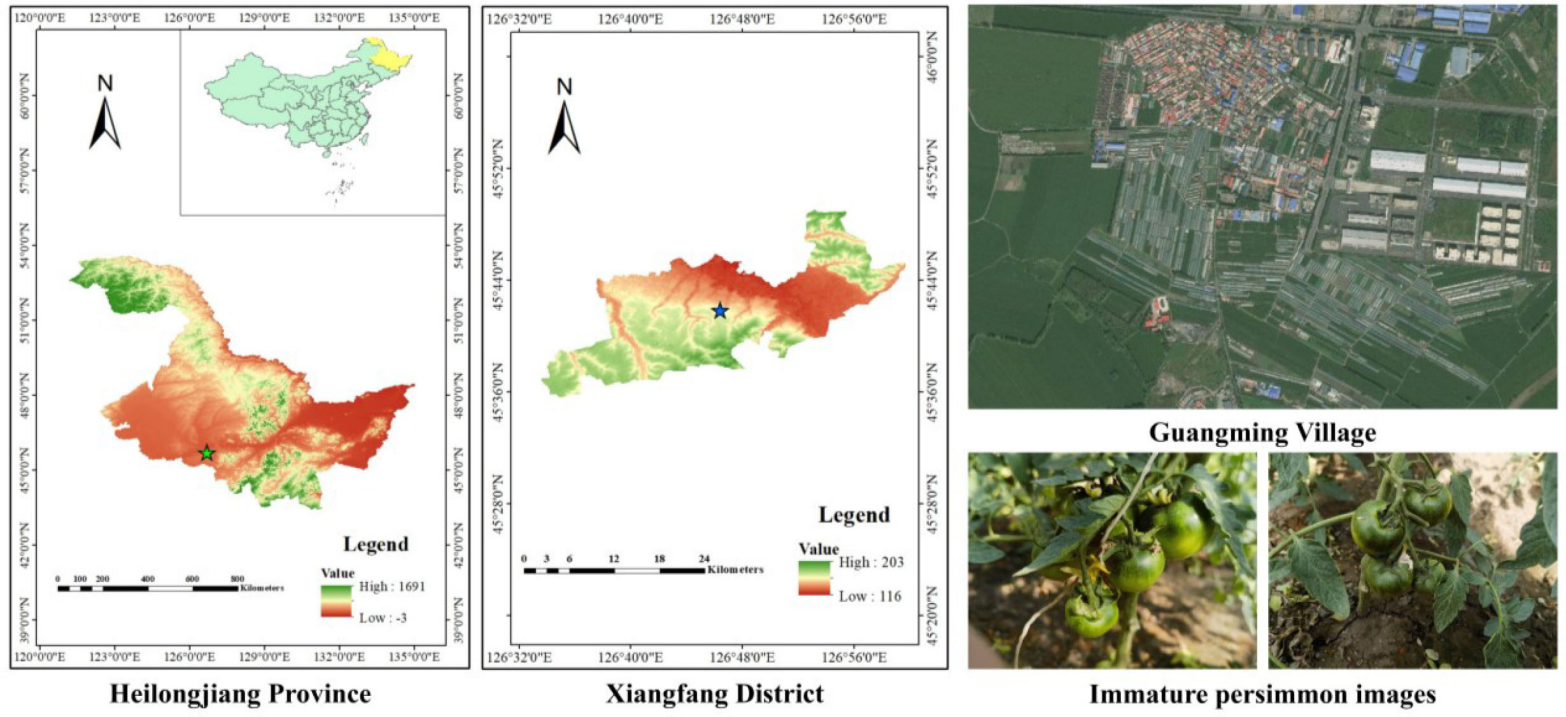
Image acquisition area for immature persimmon samples in this study.

**Figure 2:**
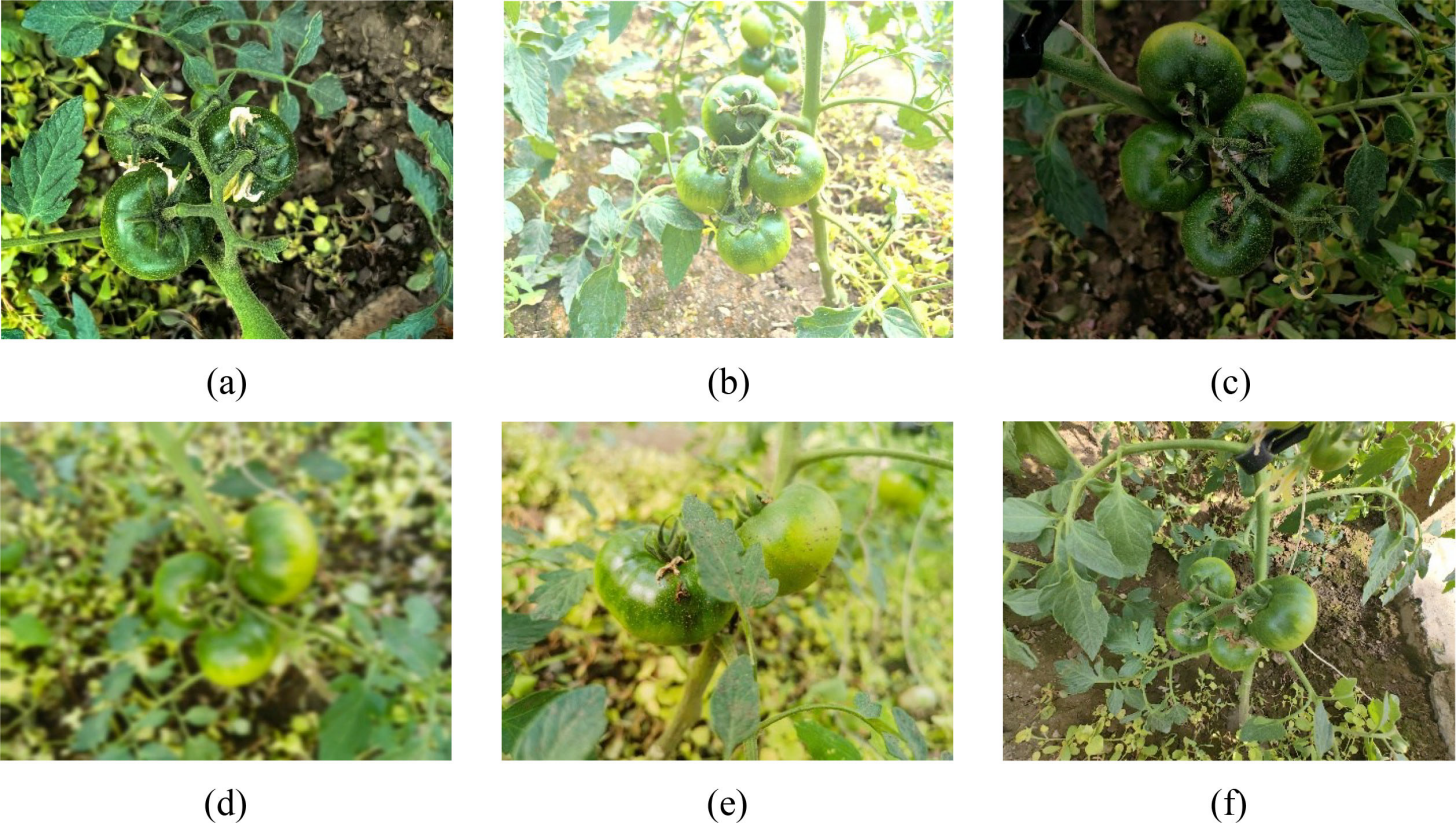
Persimmon fruit images acquired under different environmental conditions. **(a)** Branch occlusion, **(b)** Bright light, **(c)** Dark light, **(d)** Image blurring, **(e)** Leaf occlusion, **(f)** Normal light.

To ensure the reliability and representativeness of subsequent model training and validation, this study conducted image acquisition focusing on the authentic phenotypic characteristics of immature persimmons under natural growing conditions. Image collection was carried out at a fruit and vegetable production base in Guangming Village, Xingfu Town, Xiangfang District, Harbin City, Heilongjiang Province, China (126°45′50″–126°47′22″E, 45°41′06″–45°41′53″N), as illustrated in **Figure 1**. Data acquisition was performed during the critical growth stage of persimmon fruits, during which immature fruits exhibit pronounced differences in color, morphology, and texture compared with the mature stage. To enhance sample diversity and improve the robustness of the dataset, images were captured under typical field cultivation conditions. Specifically, six representative scenarios were considered: branch occlusion, bright light, dark light, image blurring, leaf occlusion, and normal light. Representative examples of these conditions are illustrated in **Figure 2**. During image acquisition, a high-resolution imaging device was employed to capture the persimmon fruits from multiple viewing angles. The shooting height and distance were dynamically adjusted according to the canopy and branch structure to ensure clear and accurate representation of immature persimmon fruits in the images. All images were stored in JPG format. Following data collection, samples exhibiting overexposure or severe defocus were manually removed to maintain consistency and quality in the original image data. Through this acquisition process, a total of 1,368 images of immature persimmons were obtained. The resulting dataset faithfully reflects the growth characteristics of immature persimmons and the complexity of real-field backgrounds, thereby providing a solid foundation for improving model performance and conducting comprehensive effectiveness evaluations.

#### 2.1.2 Data augmentation

**Figure 3:**
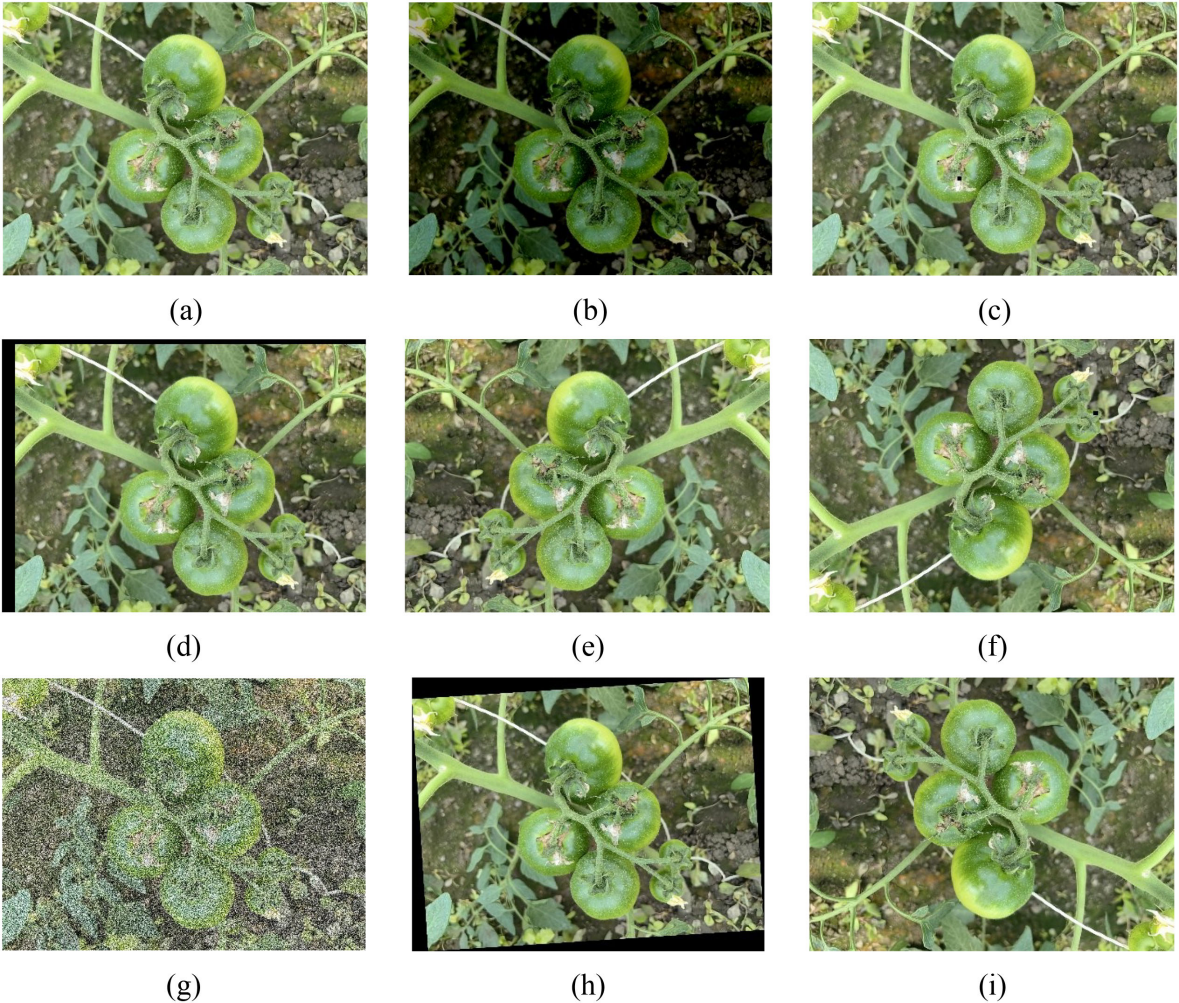
Persimmon fruit images after data augmentation. **(a)** Original image, **(b)** Brightness transformation, **(c)** Image cutting, **(d)** Image translation, **(e)** Mirror image (left and right), **(f)** Mirror image (upper and lower), **(g)** Noise addition, **(h)** Rotation angle (arbitrary angle), **(i)** Rotation angle (180°).

To mitigate the risk of overfitting caused by the uneven spatial distribution of immature persimmon samples in natural orchard environments and the complexity of imaging conditions, this study applied data augmentation techniques (Wang et al., 2024c) to preprocess the original image dataset, thereby enhancing the model’s robustness to variations in target appearance and environmental interference. Data augmentation is an image processing strategy that effectively expands the training dataset by introducing diverse geometric transformations and spectral perturbations to the original images without altering their semantic labels, which in turn improves the model’s generalization ability and robustness in complex scenes. In this study, image processing and data augmentation operations were conducted using PyCharm 2021 (JetBrains, Prague, Czech Republic). Specifically, eight augmentation methods were employed, including original image, brightness transformation, image cutting, image translation, mirror image (left and right), mirror image (upper and lower), noise addition, rotation angle (arbitrary angle), and rotation angle (180°). These augmentation strategies provide a reliable data foundation for achieving stable and high-precision recognition performance in the subsequent detection network. Through these image processing procedures, the size of the original image dataset was doubled, with representative examples illustrated in **Figure 3**.

#### 2.1.3 Dataset establishment

In this study, the open-source image annotation tool LabelImg (https://github.com/tzutalin/labelImg) was employed to annotate the ginkgo fruit image dataset. LabelImg is an interactive annotation software based on graphical interface, which offers advantages such as intuitive operation and high annotation accuracy. After running the LabelImg script, each input image is annotated individually to accurately reflect the visual characteristics of immature persimmons in real field conditions. During the annotation process, a unified annotation category, “Persimmon”, was assigned to denote immature persimmon fruits, while efforts were made to minimize the inclusion of background regions so as to reduce their potential interference with model training. After the annotation is completed, LabelImg automatically generated an XML file corresponding to each annotated image. These XML files follow the standard Pascal VOC format and contain essential information, including the image filename, resolution, object class labels, and precise coordinates of the annotated bounding boxes. This standardized annotation format provides reliable and structured input data for subsequent data parsing and model training.

### 2.2 Construction of the MCD-YOLOv12n model

#### 2.2.1 MCD-YOLOv12n network structure

The you only look once (YOLO) algorithm (Redmon et al., 2016) represents a class of object detection methods based on the idea of end-to-end single-stage detection. Target localization and category prediction are completed simultaneously within a single forward pass. This design avoids the computational redundancy caused by the separation of region proposal generation and classification-regression in traditional two-stage detection frameworks. By partitioning the input image into a set of grid cells and directly regressing bounding box parameters and class probabilities in a multi-scale feature space, YOLO effectively achieves a favorable balance between detection speed and accuracy. YOLOv12 (Tian et al., 2025) is one of the latest models in the YOLO family. Building upon the high-efficiency inference characteristics inherited from earlier YOLO variants, YOLOv12 further optimizes the backbone network and feature fusion architecture by incorporating more refined multi-scale feature interaction mechanisms. These enhancements enable the model to achieve stronger semantic representation capability while maintaining relatively low computational complexity.

**Figure 4:**
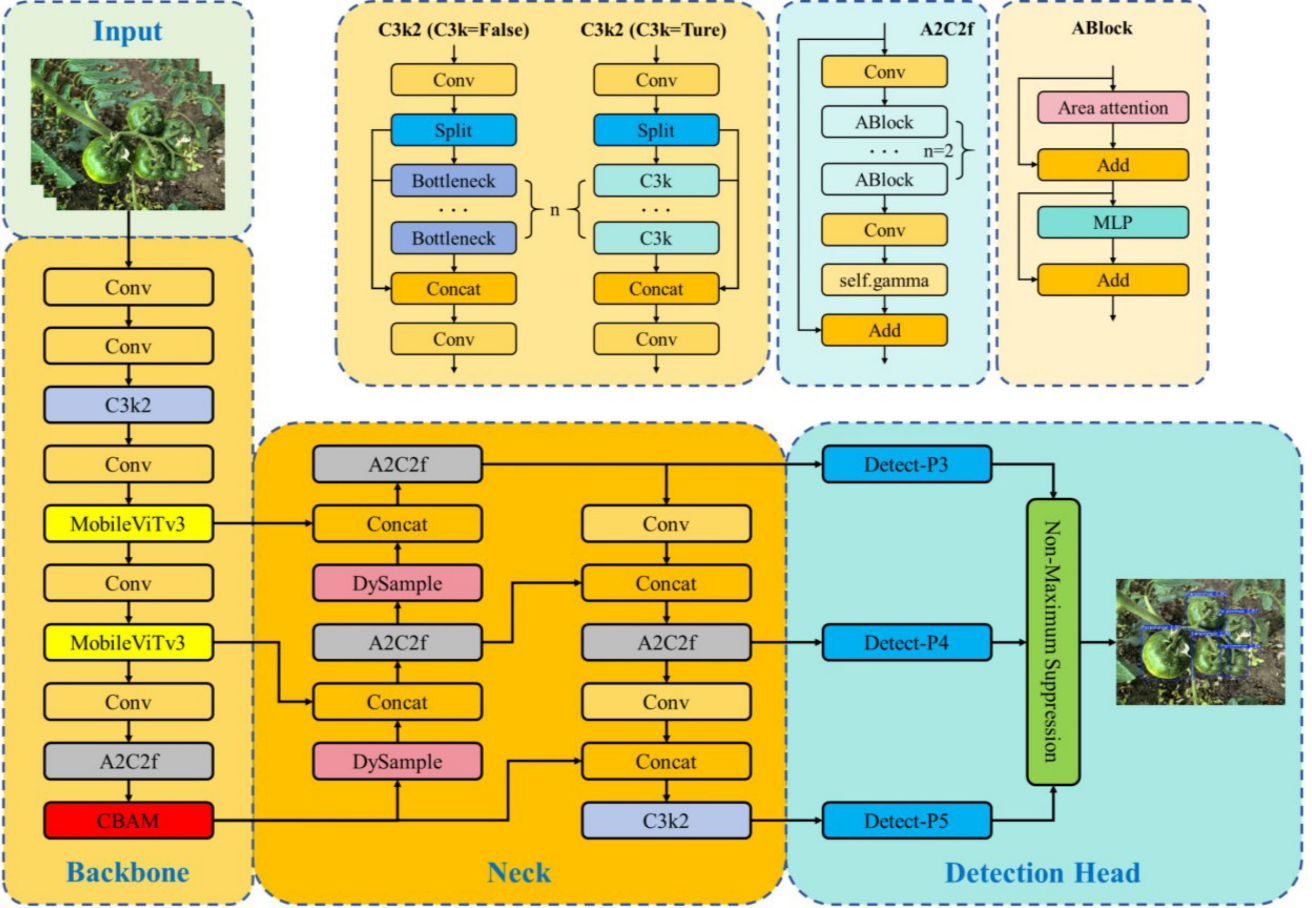
Overall network architecture of MCD-YOLOv12n.

YOLOv12 has five different network models, including YOLOv12l, YOLOv12m, YOLOv12n, YOLOv12s and YOLOv12x. The depth of the network and the width of the feature map are different. Among these variants, YOLOv12n substantially reduces the number of parameters and floating-point operations by streamlining network depth and channel width, making it particularly suitable for scenarios with limited computational resources or stringent real-time requirements (Weng et al., 2025). Considering the practical demands of field operations for high inference speed and lightweight deployment, this study adopts the YOLOv12n network architecture. Specifically, the model consists of four main components: Input, Backbone, Neck, and Detection head. At the Input stage, YOLOv12 standardizes incoming data through a unified image preprocessing pipeline. By integrating adaptive scaling and padding strategies, images of varying resolutions are resized to a fixed standard size while preserving the geometric integrity of targets, thereby enhancing the model’s robustness to scale variations. Within Backbone, a well-designed stacking of multi-layer convolutions and residual structures enables progressive modeling of low-level texture features and high-level semantic representations. This hierarchical feature extraction allows the network to effectively discriminate immature persimmon fruits from visually similar background elements, such as branches, leaves, and fruit stems, even under complex field conditions. In addition, Neck further strengthens multi-scale feature fusion by adopting a bidirectional information flow that integrates both top-down and bottom-up pathways. This design enables sufficient interaction among features at different hierarchical levels, allowing shallow spatial detail information to complement deep semantic representations, thereby significantly enhancing detection performance for small-scale and occluded targets. At the Detection head stage, YOLOv12 follows a decoupled detection paradigm in which classification and regression tasks are modeled independently. This strategy alleviates optimization conflicts caused by task coupling while efficiently producing object categories, confidence scores, and precise localization information. During the final output stage, non-maximum suppression (NMS) is applied to filter multi-scale candidate bounding boxes by evaluating their overlap ratios in conjunction with confidence scores, ensuring that only the most representative detection results are retained. In this study, the Backbone architecture of YOLOv12n is integrated with the MobileViTv3 module and the CBAM attention mechanism, while the Dysample module is incorporated into the Neck network. These enhancements collectively form the proposed MCD-YOLOv12n architecture. The overall structure of the MCD-YOLOv12n model is illustrated in **Figure 4**.

#### 2.2.2 MobileViTv3

**Figure 5:**
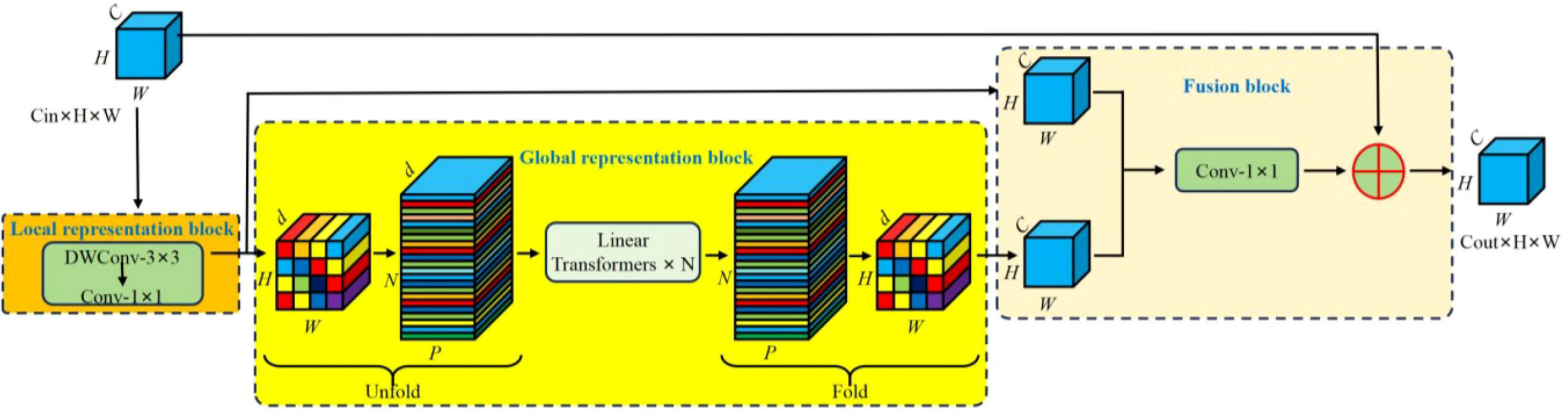
MobileViTv3 module structure diagram.

MobileViTv3, proposed by Wadekar et al (Wadekar and Chaurasia, 2022), is an efficient vision module that deeply integrates lightweight Transformers with CNNs. Its core principle is to introduce self-attention mechanisms to enhance global feature representation while maintaining a controllable number of parameters and computational complexity. Accordingly, in this study, the MobileViTv3 module (see **Figure 5**) is embedded into the Backbone feature extraction stage of YOLOv12 to achieve collaborative optimization between local detail perception and global semantic modeling. This integration effectively improves the recognition accuracy and robustness of immature persimmons under complex background conditions. Let the input feature map be denoted as $X\in {\mathbb{R}}^{H\times W\times C}$, where H, *W*, and *C* represent its height, width, and number of channels, respectively. First, local feature representations are extracted from the input feature map using a standard 3×3 depthwise separable convolution. Subsequently, a 1×1 convolution is employed to perform a linear projection along the channel dimension, thereby reducing the computational burden of the subsequent Transformer module. To incorporate global modeling capability, feature map $X_{p}$ is spatially partitioned into a set of non-overlapping patches $\left\{X_{1},X_{2},\cdots ,X_{N}\right\}$. Here, $N=\frac{H\times W}{P^{2}}$, and *P* denotes the patch size. Each patch is flattened and then linearly projected into a token representation. During the Transformer encoding stage, global dependency relationships are captured using a multi-head attention mechanism, whose computation can be formulated in (Equation 1):

(1) $$Attention(Q,K,V)=Soft\max(\frac{QK}{\sqrt{d}})V$$

Where $Q=ZW_{Q}$, $K=ZW_{K}$, and $V=ZW_{V}$, $Z\in {\mathbb{R}}^{N\times (P_{h}P_{w})\times D}$, $P_{h}$ denotes the height of the patch, while $P_{w}$ represents the width of the patch. $W_{Q},W_{K},W_{V}\in {\mathbb{R}}^{D\times D}$, correspond to the linear projection dimensions in the multi-head attention mechanism. Softmax(·) denotes the normalization process applied to the patch along the last dimension. The output of the multi-head attention mechanism can be expressed as follows (Equation 2):

(2) $$MHSA(Z)=Concat(head_{1},\cdots ,head_{h})W_{O}$$

Where MHSA(·) denotes the processing operation of the multi-head attention mechanism. Then, a feed-forward network (FFN) is employed to further enhance the nonlinear representational capacity of the extracted features (Equation 3):

(3) Z'=FFN(MHSA(Z))+Z

Here, Z' denotes the output representation. Subsequently, the features produced by the Transformer are remapped back to a two-dimensional spatial structure, and the channel dimensionality is restored via a 1×1 convolution. Finally, the global feature representation $X_{g}$ is integrated with the local convolutional feature $X_{l}$ to produce the final output *Y* (Equation 4).

(4) $$Y=X_{g}⊕X_{l}$$

Where, ⊕ denotes either the feature concatenation operation or the element-wise addition operation.

#### 2.2.3 CBAM

**Figure 6:**
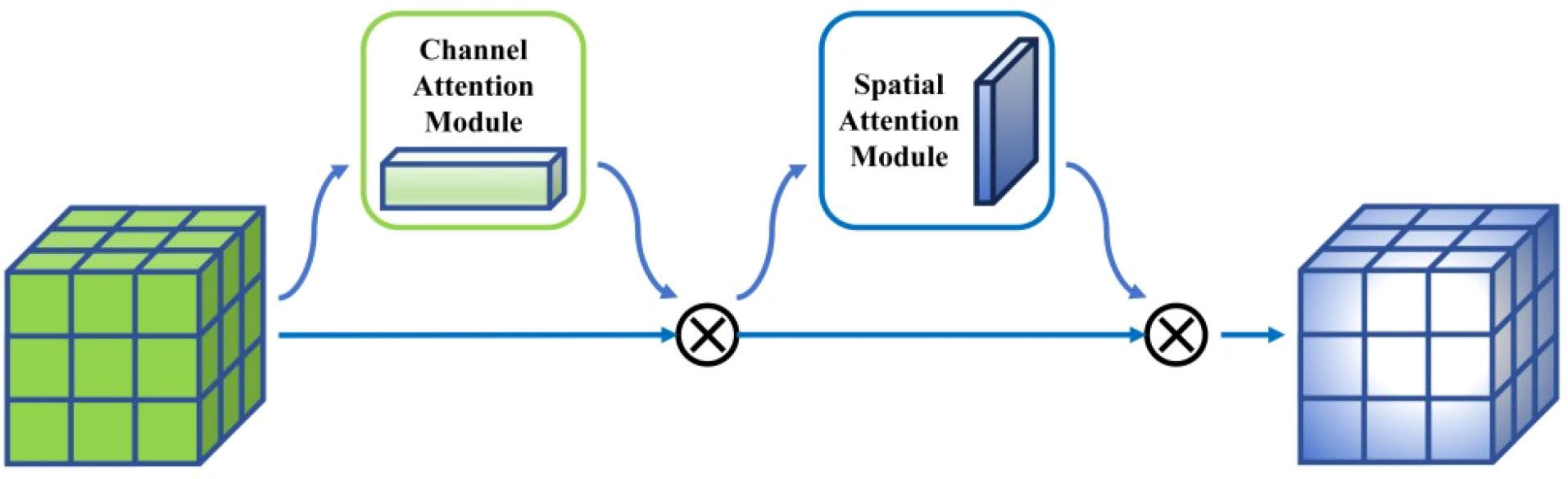
CBAM module structure diagram.

In the actual field environment for real-time recognition, immature persimmons lack obvious texture and color features in visible light images. As a result, traditional CNNs are prone to interference from complex background noise during feature extraction, which degrades their discriminative capability. To enhance the model’s ability to focus on key regions and capture discriminative features, this study incorporates the convolutional block attention module (CBAM) (Woo et al., 2018) into the YOLOv12 network architecture, enabling adaptive feature reweighting along both the channel and spatial dimensions. CBAM is a lightweight serial attention mechanism designed to improve feature representation without significantly increasing the number of model parameters. The specific structure is shown in **Figure 6**. Its core principle lies in guiding the network to emphasize informative target-related features while suppressing redundant or irrelevant information. Specifically, given an input feature map $F\in {\mathbb{R}}^{C\times H\times W}$, where C, H, and W denote the number of channels, height, and width, respectively, CBAM sequentially applies channel attention and spatial attention to generate intermediate feature maps F' and F". The overall process can be expressed in (Equations 5, 6):

(5) $$F'={\text{M}}_{c}(F)⊗F$$

(6) $$F"={\text{M}}_{s}(F')⊗F'$$

Where, ${\text{M}}_{c}(\cdot )$ and ${\text{M}}_{s}(\cdot )$ denote the channel attention map and the spatial attention map, respectively, while ⊗ represents element-wise multiplication. In the channel attention stage, CBAM applies global average pooling (GAP) and global max pooling (GMP) to compress the feature maps along the spatial dimension. This operation captures complementary channel-wise statistical information and produces two distinct channel descriptor vectors, denoted as $f_{avg}^{c}$ and $f_{\max}^{c}$ (Equation 7):

(7) $$f_{avg}^{c}=\text{GAP}(F),f_{\max}^{c}=\text{GMP}(F)$$

Subsequently, a shared multi-layer perceptron (MLP) is employed to perform nonlinear mapping, and the specific procedure can be expressed in (Equation 8):

(8) $${\text{M}}_{c}(F)=\sigma (W_{1}(W_{0}f_{avg}^{c}))+W_{1}(W_{0}f_{\max}^{c})$$

Where, $W_{0}$ and $W_{1}$ denote learnable parameters, and σ(·) represents the Sigmoid activation function. This mechanism adaptively assigns weights to different feature channels, enabling the model to place greater emphasis on channels that are highly correlated with the morphological characteristics, edge information, and spectral responses of immature persimmons, thereby enhancing feature discrimination and representation capability. In the spatial attention stage, CBAM compresses feature representations along the channel dimension. Specifically, average pooling and max pooling are applied independently to generate spatial descriptor maps $f_{avg}^{s}$ and $f_{\max}^{s}$ (Equation 9).

(9) $$f_{avg}^{s}={\text{AvgPool}}_{c}(F'),f_{\max}^{s}={\text{MaxPool}}_{c}(F')$$

Subsequently, the two feature maps are concatenated along the channel dimension and fed into a convolutional layer with a kernel size of 7×7 to generate the spatial attention weights ${\text{M}}_{s}(F')$ (Equation 10):

(10) $${\text{M}}_{s}(F')=\sigma (f^{7\times 7}([f_{avg}^{s};f_{\max}^{s}]))$$

This process highlights the spatial distribution of potential target regions within the image, thereby enhancing the model’s ability to perceive fruit contours as well as locally salient regions in a spatially aware manner.

#### 2.2.4 Dysample

**Figure 7:**
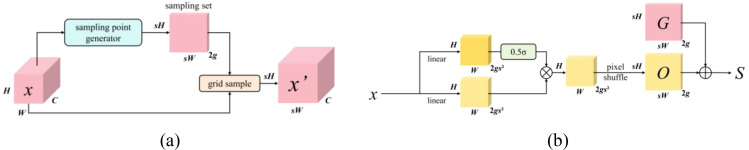
Dysample module structure diagram. **(a)** Sampling based dynamic upsampling, **(b)** Sampling point generator in DySample.

In the task of immature persimmon fruit recognition, significant challenges commonly arise, including large scale variations, blurred boundaries, severe occlusion, and background colors that are highly similar to the target objects. These factors impose higher demands on the spatial recovery capability of multi-scale features within the detection network. In the classical YOLOv12 framework, commonly adopted nearest-neighbor or bilinear upsampling methods rely on fixed interpolation rules, making it difficult to adaptively adjust according to the true spatial structure and semantic characteristics of the fruit. As a result, these approaches are prone to information loss or boundary misalignment, particularly in regions containing small-scale immature persimmon fruits. To address these limitations, this study introduces the DySample module (Liu et al., 2023) into the feature fusion stage of the YOLOv12 architecture, as illustrated in **Figure 7**. By replacing fixed upsampling operators with a learnable dynamic sampling mechanism, DySample enhances the detection accuracy of immature persimmons in complex orchard environments. Specifically, unlike traditional dynamic convolution kernel reassembly methods, the core idea of DySample is to reformulate the upsampling process from a point-sampling perspective. Given an input feature map $X\in {\mathbb{R}}^{C\times H\times W}$, DySample first resamples it using a learnable sampling set *S*, yielding the following result (Equation 11):

(11) X'=GridSample(X,S)

Where $X'\in {\mathbb{R}}^{C\times sH\times sW}$ denotes the upsampled feature map, *s* represents the upsampling scale factor, and $S\in {\mathbb{R}}^{2\times sH\times sW}$ refers to the set of sampling points, where the dimension 2 corresponds to the horizontal and vertical components of the spatial coordinates. The sampling point set *S* is jointly determined by the basic rule network *G* and the learnable offset *O*, which can be expressed in (Equation 12):

(12) S=G+O

Where, *G* denotes the initial sampling value obtained via standard bilinear interpolation, while *O* represents the dynamically predicted offset generated by the network, which is employed to characterize local semantic–driven spatial adjustments. To prevent excessive offset magnitudes that could lead to sampling point overlap and semantic distortion, Dysample imposes adaptive constraints on the predicted offsets. The generation of these constrained offsets is defined in (Equation 13):

(13) $$O=0.5\cdot \sigma (Conv_{1}(X))⊙Conv_{2}(X)$$

Here, $Conv_{1}(\cdot )$ and $Conv_{2}(\cdot )$ denote two 1×1 convolutional operators, σ(·) represents the Sigmoid function, which generates dynamic scaling factors with values constrained to the range (0, 1), and ⊙ denotes element-wise multiplication. Furthermore, to enhance the model’s capability to characterize semantic information across different channels, Dysample introduces a grouping mechanism along the channel dimension. Within this design, each group of channels shares a common set of sampling offsets, enabling the parallel reconstruction of multiple semantic subspaces while effectively reducing both the number of parameters and the computational complexity.

### 2.3 Ablation experiment

To systematically verify the underlying mechanisms and necessity of each improved module in the task of immature persimmon detection, this study conducts an ablation study (Wang et al., 2024b) to comparatively analyze the baseline model and its variants with different combinations of improvements. An ablation study is a widely adopted analytical approach in which individual functional modules of a model are progressively removed, replaced, or added to quantify the contribution of each component to the overall performance. The primary objective of this method is to elucidate the intrinsic sources of performance gains, making it a crucial tool for interpreting and optimizing complex neural network architectures. Owing to its effectiveness, ablation analysis has been extensively employed in recent studies (Lu et al., 2025; Yi et al., 2025). In this work, the original YOLOv12 network is selected as the baseline model, and a series of comparative experiments are designed by incrementally introducing and jointly configuring the MobileViTBv3, CBAM, and DySample modules. The ablation models and their corresponding module configurations are shown in **Table 1**.

**Table 1:** Improved models in the ablation experiment and their corresponding modules.

Improved models	Improved modules			
	YOLOv12n architecture	MobileViTBv3	CBAM	Dysample
YOLOv12n	✓	—	—	—
Case1	✓	✓	—	—
Case2	✓	—	✓	—
Case3	✓	—	—	✓
Case4	✓	✓	✓	—
Case5	✓	✓	—	✓
Case6	✓	—	✓	✓
MCD-YOLOv12n	✓	✓	✓	✓

### 2.4 Software and hardware configuration

To ensure the reliability and comparability of the experimental results, all models were trained and evaluated under identical hardware and software conditions. The experiments were conducted on a computing platform equipped with an Intel^®^ Core™ i7-14700KF processor operating at a base frequency of 3.4GHz, 16GB of DDR5 system memory, and an NVIDIA GeForce RTX 4060 Ti graphics processing unit. On the software side, an accelerated computing environment was established using CUDA 11.8 and cuDNN 8.6, and all experiments were implemented in Python 3.8 with the PyTorch 1.13.1 deep learning framework. During training, the initial learning rate was set to 0.01 to ensure stable parameter updates, while a weight decay coefficient of 0.0005 was introduced to mitigate the risk of overfitting. A momentum value of 0.937 was adopted to enhance gradient optimization efficiency. Each model was trained for 200 epochs with a batch size of 8, achieving a balanced trade-off between training efficiency and convergence performance.

### 2.5 Model evaluation

In performance evaluation of object detection models, comprehensive metrics are essential for validating both model effectiveness and practical applicability. For the task of immature persimmon fruit recognition, which represents a typical and challenging detection problem in complex natural field environments, this study adopts precision, recall, mAP, and F1-score as the primary performance evaluation metrics. In addition, this study employs parameters, FLOPs, model size, and frame per second (FPS) to assess the computational complexity and deployment efficiency of the proposed ESE-YOLOv11n model. The corresponding formulations of these metrics are presented in (Equations 14–17).

(14) $$Precision_{i}=\frac{x_{ii}}{\sum_{j=1}^{n}x_{ij}}$$

(15) $$Recall_{j}=\frac{x_{jj}}{\sum_{i=1}^{n}x_{ij}}$$

(16) $$mAP=\frac{1}{n}\sum_{i=1}^{n}\int_{0}^{1}Precision_{i}d(Recall_{i})$$

(17) $$F1-Score_{i}=\frac{2\times Precision_{i}\times Recall_{i}}{Precision_{i}+Recall_{i}}$$

Where *n* denotes the total number of classes; *i* = 1, 2, 3, …, *n*, represents the class index of the ground-truth samples; and *j* = 1, 2, 3, …, *n*, denotes the class index of the predicted samples. $x_{ij}$ indicates the number of samples in the test set whose true class is *i* but are incorrectly predicted as class *j*, while $x_{ii}$ denotes the number of samples in the test set whose true class and predicted class are both *i*.

## 3 Results and discussion

### 3.1 Visual analysis of different improved models under diverse environmental conditions

**Figure 8:**
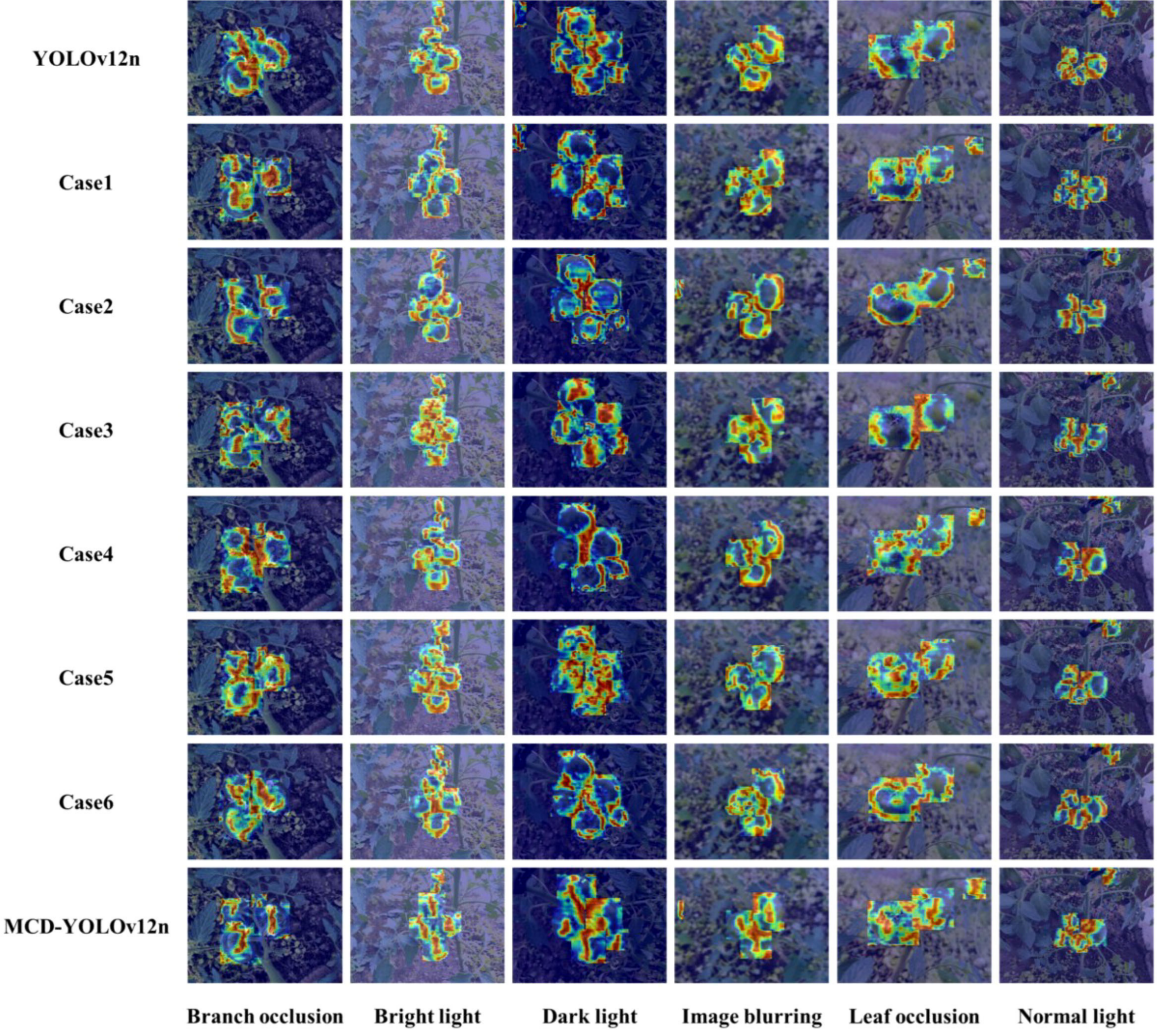
Visualization results of different improved models under diverse environmental conditions.

This study employs the gradient-weighted class activation mapping (Grad-CAM) technique (Selvaraju et al., 2016) to validate the rationality of the discriminative basis underlying the MCD-YOLOv12n model. Grad-CAM is a gradient-based visualization technique. Its core idea is to exploit the gradients of a target class with respect to high-level convolutional feature maps in the network. These gradients are used to compute the contribution weight of each channel to the final prediction. Based on these weights, a spatial response heatmap is generated to highlight class-discriminative regions. By overlaying the heatmap onto the original image, Grad-CAM enables a clear depiction of the key regions emphasized by the model during category discrimination, providing an intuitive interpretation of the model’s decision-making process. In the Grad-CAM heatmaps, different colors indicate varying levels of model attention. Specifically, red or dark red regions denote areas assigned high response weights by the network and typically correspond to the fruit body or its prominent contour features. Yellow and orange regions represent features of moderate importance, suggesting that these areas play an auxiliary role in the recognition process. In contrast, cooler colors such as green and blue indicate regions receiving relatively low attention, which are generally associated with background areas or components weakly related to target discrimination. The Grad-CAM visualization results obtained in this study are presented in **Figure 8**.

**Figure 8** illustrates the Grad-CAM visualizations of different models under a variety of complex environmental conditions, revealing distinct attention distribution patterns. For the baseline YOLOv12 model, under normal light conditions, the red and dark-red activation regions are generally able to cover the main body of the persimmon fruit. However, the attention distribution remains relatively scattered, with noticeable discontinuities between the fruit edges and internal regions. In scenarios involving branch occlusion and leaf occlusion, the high-response regions of this model are significantly disturbed, with some red activation areas shifting toward branches or leaf veins, leading to a degradation in the integrity of the fruit contour. These observations indicate that the baseline YOLOv12 model is unable to effectively extract discriminative features from persimmon fruits. In comparison, Case 1 demonstrates a certain degree of improvement across multiple environments. Under normal lighting and mild occlusion conditions, the high-response regions are more closely aligned with the fruit body. However, there is still a problem of attention shifting to local bright spots or shadow areas in strong light and weak light environments. By incorporating CBAM, Case 2 further enhances attention to fruit edges and key structural features under branch and leaf occlusion, where yellow and orange regions provide effective reinforcement of the fruit contour. However, localized over-activation is still observed under strong light conditions, indicating that excessive responses in high-intensity regions have not been completely eliminated. Case 3 demonstrates relatively strong scale adaptability under image blurring and dark light conditions, with more complete high-response regions over the fruit body. However, it remains somewhat sensitive to branches under severe occlusion. Compared with Cases 1–3, the multi-module configurations in Cases 4–6 exhibit a more stable overall attention distribution across diverse environments, accompanied by improved continuity of responses over the fruit body. This indicates that multi-module collaboration enhances feature representation to a certain extent. Notably, MCD-YOLOv12n achieves the most favorable visualization performance across all scenarios. Its red and dark-red high-response regions are highly concentrated on the immature persimmon fruit itself, yielding clear contours and strong consistency. Moreover, it effectively suppresses background interference under branch occlusion, leaf occlusion, bright light, dark light, and image blurring conditions. Overall, the Grad-CAM visualizations robustly confirm that MCD-YOLOv12n possesses superior discriminative capability for key features of immature persimmons in complex natural environments, significantly outperforming the baseline YOLOv12 model as well as other improved variants.

### 3.2 Comparison of detection results of different models under varying environmental conditions

To systematically evaluate the detection performance of the proposed MCD-YOLOv12n model for immature persimmon fruits in complex scenes, this study conducted a comprehensive comparative analysis among the baseline YOLOv12n, Case1–Case6 variants, and MCD-YOLOv12n, with particular emphasis on examining the interactions among individual modules. To ensure the reliability and fairness of the experimental results, all models were trained and tested under identical operating environments and hyperparameter configurations. The comparative results are presented in **Table 2**.

**Table 2:** Detection results of persimmon fruits achieved by different improved models under various environmental conditions.

Models	Precision (%)	Recall (%)	mAP (%)	FLOPs	Parameters	Weight size (MB)	FPS
YOLOv12n	91.50	92.40	92.50	6.0G	2,538,486	5.22	91.74
Case1	92.60	88.90	86.20	8.8G	2,801,302	5.72	81.75
Case2	93.90	91.10	94.60	6.0G	2,555,612	5.25	83.33
Case3	93.50	89.70	94.40	6.0G	2,550,838	5.24	87.74
Case4	94.60	91.40	95.00	13.3G	3,654,460	7.36	69.44
Case5	94.30	89.00	94.70	8.8G	2,813,654	7.39	63.69
Case6	95.50	90.80	94.80	10.5G	3,403,996	6.88	52.63
MCD-YOLOv12n	96.30	89.90	95.30	13.3G	3,666,812	7.39	72.5

The results presented in **Table 2** indicate that all models achieve satisfactory detection performance, with precision values exceeding 90%. However, notable differences are observed among the models in terms of detection accuracy, computational efficiency, and model complexity. The baseline YOLOv12 model attains precision and recall values of 91.50% and 92.40%, respectively, while maintaining relatively low parameter counts and computational costs. This suggests that the model is highly practical under normal light conditions and scenarios with clear target features. However, it still faces risks of missed detections and false positives in complex backgrounds, under occlusion, or in varying lighting conditions. Compared with the baseline, Case1 exhibits an improvement in precision while maintaining unchanged FLOPs, parameter counts, and weight size. However, its recall and mAP decrease noticeably, indicating that the structural modification provides limited stability in extracting critical features under complex environmental conditions. In Case 2, the introduction of the CBAM attention mechanism significantly improves the mAP while maintaining a low computational cost. This result indicates that CBAM can more effectively highlight the discriminative features of immature persimmons under conditions of branch and leaf occlusion as well as background interference. Case3 shows improved adaptability in scenarios involving blur and scale variations, maintaining a relatively high mAP while effectively controlling model complexity. Case4 and Case5 further improve detection accuracy and mAP through multi-module integration, suggesting that collaborative feature representation enhances discriminative capability in complex environments. However, this performance gain is accompanied by a certain degradation in real-time recognition efficiency. Case6 achieves one of the highest recognition accuracies among the improved models, second only to the proposed MCD-YOLOv12n, but records the lowest inference speed at 52.63FPS. Notably, MCD-YOLOv12n achieves an effective balance between detection speed and accuracy across diverse complex conditions, with precision and mAP reaching 96.30% and 95.30%, respectively, while maintaining robust performance under bright light, dark light, occlusion, and image blurring. Moreover, its inference speed of 72.5FPS far exceeds the real-time requirements of practical applications, fully demonstrating the comprehensive superiority of MCD-YOLOv12n for immature persimmon detection tasks.

**Figure 9:**
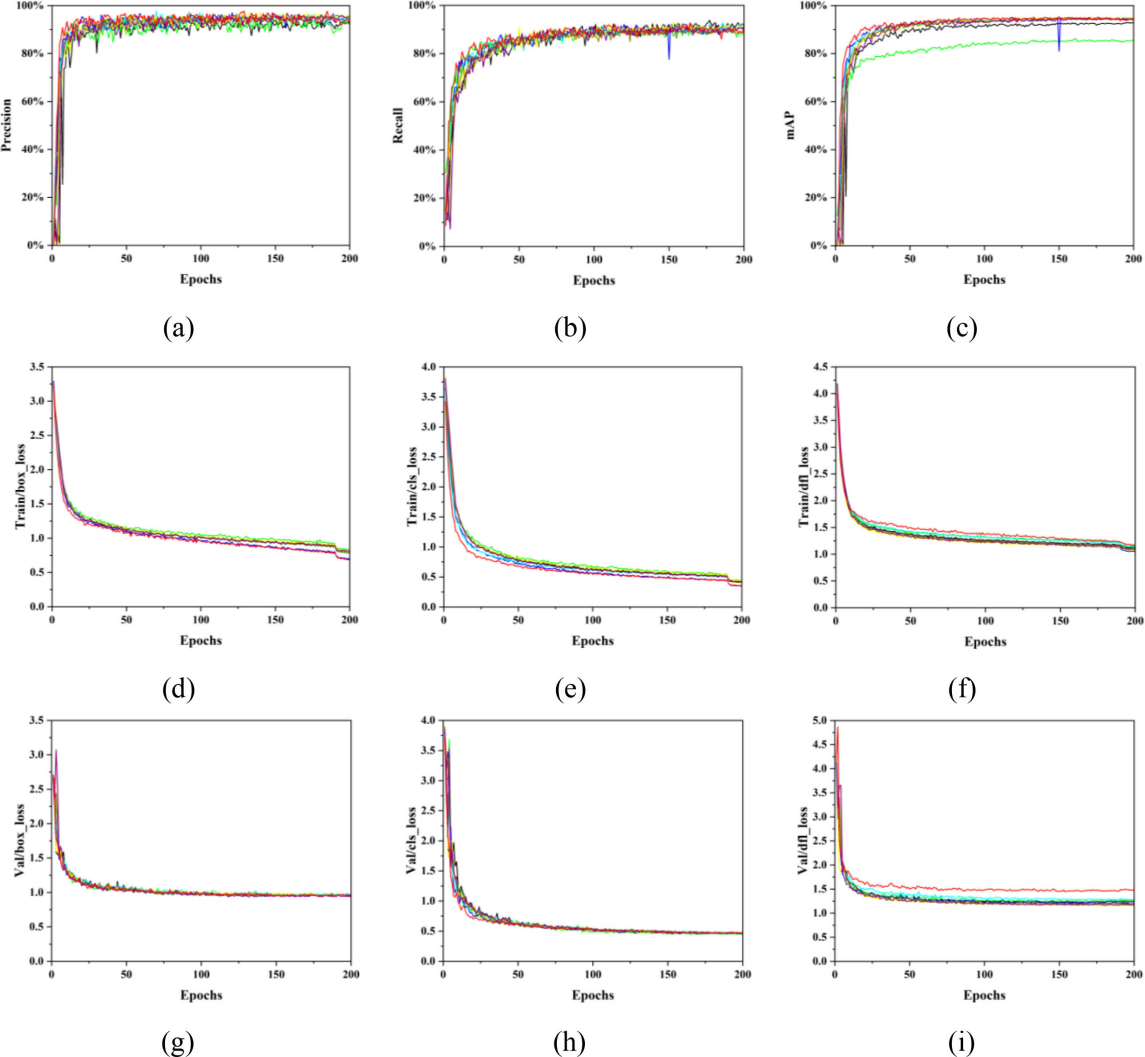
Performance curves of different improved network models. **(a)** Precision, **(b)** Recall, **(c)** mAP, **(d)** Boundary box loss of training set, **(e)** Classification loss of training set, **(f)** Confidence loss of training set, **(g)** Boundary box loss of validation set, **(h)** Classification loss of validation set, **(i)** Confidence loss of validation set. Different colors are used to represent different models: ■ YOLOv12n, ■ Case1, ■ Case2, ■ Case3, ■ Case4, ■ Case5, ■ Case6, ■ MCD-YOLOv12n.

**Figure 9** illustrates the training dynamics of all models, showing that precision, recall, and mAP increase initially and then gradually stabilize, while the loss curves exhibit a decreasing trend followed by convergence. In the precision and recall curves, the performance differences among the models are not distinctly separable. Nevertheless, it can be observed that the baseline YOLOv12n model consistently yields the lowest values and exhibits the largest fluctuations, indicating inferior stability and performance. This suggests that the integration of different modules in the proposed variants effectively enhances overall model performance. In the recall curve, Case2 demonstrates the most prominent behavior, exhibiting a sharp decrease followed by a rapid increase around 150 iterations. However, the final converged recall value of this model is not the lowest among all the compared models. Compared with the precision and recall curves, the mAP curve provides a clearer distinction between different models. Notably, Case1 achieves a significantly lower mAP than all other models, indicating insufficient global discriminative capability and limited localization robustness. Similar to the recall curve, the mAP of Case2 also shows a sudden drop and subsequent rise around 150 iterations. Overall, the proposed MCD-YOLOv12n model attains the highest converged mAP value, followed by Case6, while the classical YOLOv12n model performs only slightly better than Case1. Regarding training losses, clear differences can be observed among all models. The MCD-YOLOv12n model achieves the lowest localization and classification losses, whereas its regression loss is the highest. In contrast, the validation losses for localization and classification do not exhibit clear differences across models. It is noteworthy that, in terms of validation regression loss, MCD-YOLOv12n again presents the highest value. This indicates that there remains room for improvement in the model’s target localization and scale regression capability, and future work could further enhance performance by incorporating optimized or hybrid loss functions.

### 3.3 Comparison of results with classical models under different environmental conditions

**Figure 10:**
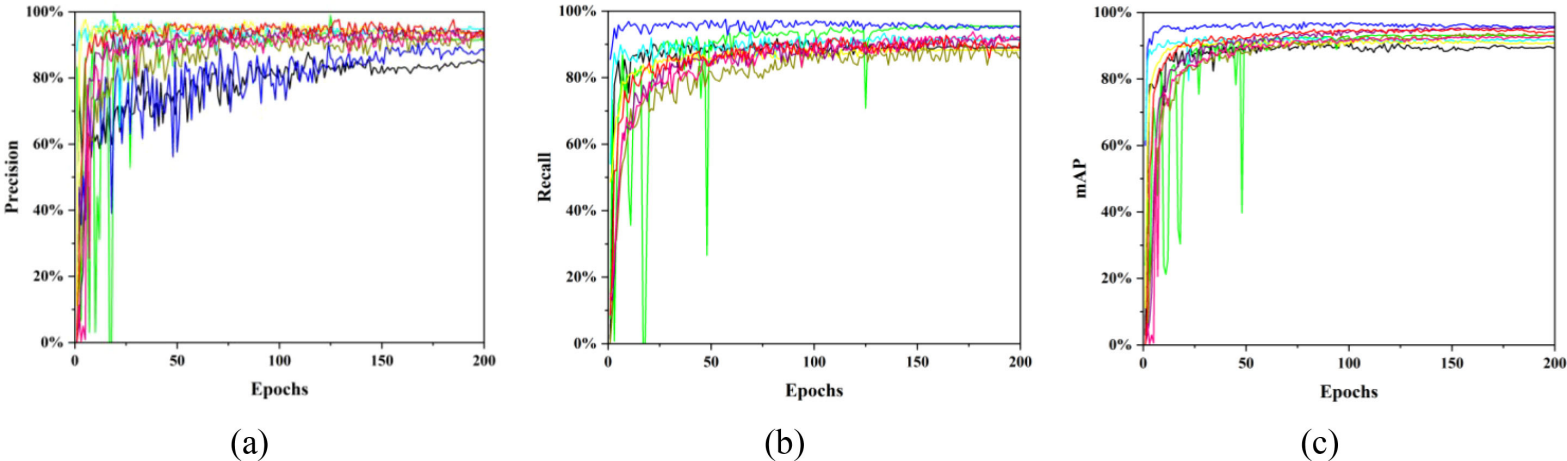
Comparative performance curves between MCD-YOLOv12n and classical detection models. **(a)** Precision, **(b)** Recall, **(c)** mAP. Different colors represent different models: ■ DETR, ■ EfficientDet, ■ Faster R-CNN, ■ RetinaNet, ■ SSD, ■ YOLOv10, ■ YOLOv11n, ■ YOLOv12n, ■ MCD-YOLOv12n.

To further validate the detection performance of MCD-YOLOv12n for identifying immature persimmon fruits, this study compares its detection results on a persimmon fruit image dataset with those of several representative and widely adopted methods in the current field of object detection, including DETR, EfficientDet, Faster R-CNN, and YOLO series algorithms (Rehman et al., 2019; Zhao et al., 2024). These methods represent mainstream models from different developmental stages and collectively reflect the evolutionary trend in the performance of object detection approaches. To ensure the fairness and comparability of the experimental results, MCD-YOLOv12n and all comparative models were trained and evaluated under identical conditions, using the same input resolution and an entirely consistent experimental environment. The comparative performance results are presented in **Table 3** and **Figure 10**.

**Table 3:** Comparison of detection results between MCD-YOLOv12n and classical deep learning models.

Models	Precision (%)	Recall (%)	mAP (%)	F1-score	Weight size (MB)	FPS
DETR	83.17	88.11	89.77	0.86	158.89	6.72
EfficientDet	90.07	95.13	93.05	0.93	25.63	6.64
Faster R-CNN	86.16	94.81	95.88	0.90	108.17	4.49
RetinaNet	93.19	90.59	91.11	0.92	138.91	11.93
SSD	92.36	89.26	93.15	0.91	90.61	32.41
YOLOv10n	90.60	89.90	93.70	0.90	5.48	63.69
YOLOv11n	93.10	93.00	95.40	0.93	5.22	39.20
YOLOv12n	91.50	92.40	92.50	0.92	5.27	91.74
MCD-YOLOv12n	96.30	89.90	95.30	0.93	7.39	62.50

As shown in the results presented in **Table 3**, the evaluated detection models exhibit noticeable differences in both recognition accuracy and inference speed. Specifically, the Transformer-based DETR demonstrates a certain degree of global modeling capability in complex backgrounds. However, its detection performance is the weakest among all evaluated models, with precision, recall, and mAP values of only 83.17%, 88.11%, and 89.77%, respectively. Meanwhile, the model exhibits a weight size of 158.89MB and an inference speed of only 6.72FPS, indicating that it is unsuitable for real-time fruit recognition in complex field environments. EfficientDet shows relatively strong performance in terms of recall, reflecting its robust target coverage capability across diverse field scenes, but its relatively large model size and limited FPS still restrict its applicability in real-time scenarios. Faster R-CNN exhibits strong feature representation capability, achieving recall and mAP values of 94.81% and 95.88%, respectively. However, its inference speed is relatively low, reaching only 4.99FPS, which limits its applicability in real-time scenarios. RetinaNet achieves the highest precision among all classical models, suggesting its suitability for the detection of immature persimmon fruits. In contrast, the YOLO-series models demonstrate a more favorable balance between accuracy and speed. Under lightweight configurations, YOLOv10n and YOLOv11n achieved inference speeds of 63.69FPS and 39.20FPS, respectively. However, in complex field environments, further improvements in detection accuracy remain constrained by the inherent limitations of their network architectures. YOLOv12n introduces enhancements in multi-scale feature fusion, boosting the inference speed to 91.74FPS. However, its precision and mAP are limited to 91.50% and 92.50%, respectively. Notably, the proposed MCD-YOLOv12n model achieves a precision of 96.30% and an mAP of 95.30% while maintaining a relatively compact model size and a real-time inference speed of 62.50FPS. These results demonstrate that the proposed model offers significant advantages in terms of accuracy, real-time performance, and practical feasibility, making it more suitable for the real-world field identification of immature persimmon fruits.

**Figure 10** illustrates the training dynamics of different models on the immature persimmon fruit dataset. During training, the precision, recall, and mAP curves of all models exhibit similar overall trends, the metrics gradually increase to their maximum values and then fluctuate slightly around the peak. Compared with the recall and mAP curves, the precision curves show the largest oscillation amplitudes. Among all models, Faster R-CNN and DETR de monstrate the most pronounced fluctuations, indicating relatively poor robustness and a limited ability to effectively cope with variations in complex field environments. In contrast, the YOLO-based models exhibit the highest stability, further confirming their superior robustness. Among all evaluated models, MCD-YOLOv12n achieves the highest final convergence precision, followed by YOLOv11, while Faster R-CNN and DETR show the lowest convergence precision. In the recall curves, Faster R-CNN attains the highest final convergence value and maintains relatively stable performance throughout training. The EfficientDet model ranks second in terms of final recall. However, it exhibits the largest oscillations, with the recall dropping abruptly to 0% at the 18th iteration before rapidly recovering. A similar phenomenon is observed in the mAP curves, where sudden decreases followed by sharp increases are also evident. Moreover, Faster R-CNN achieves the highest final converged mAP among all models, demonstrating strong discriminative capability. The mAP curves further reveal a stratification in the final convergence values, allowing effective differentiation among the evaluated models. Specifically, the final mAP of MCD-YOLOv12n is second only to that of Faster R-CNN, whereas DETR and SSD exhibit the lowest mAP values. These observations are consistent with the results reported in **Table 3**.

### 3.4 Comparative analysis of recognition results between MCD-YOLOv12n and classical models under different environmental conditions

To comprehensively evaluate the applicability and robustness of MCD-YOLOv12n in complex real-world orchard operation scenarios, a comparative analysis was conducted under six representative environmental conditions: branch occlusion, bright light, dark light, image blurring, leaf occlusion, and normal light. The performance of several mainstream object detection models in the task of immature persimmon identification was systematically assessed. The comparison models included, SSD, RetinaNet, and multiple lightweight variants of the YOLO series. This experimental design aims to rigorously validate the effectiveness of the introduced multi-scale feature fusion and detail-aware perception mechanisms in complex environments.

**Figure 11:**
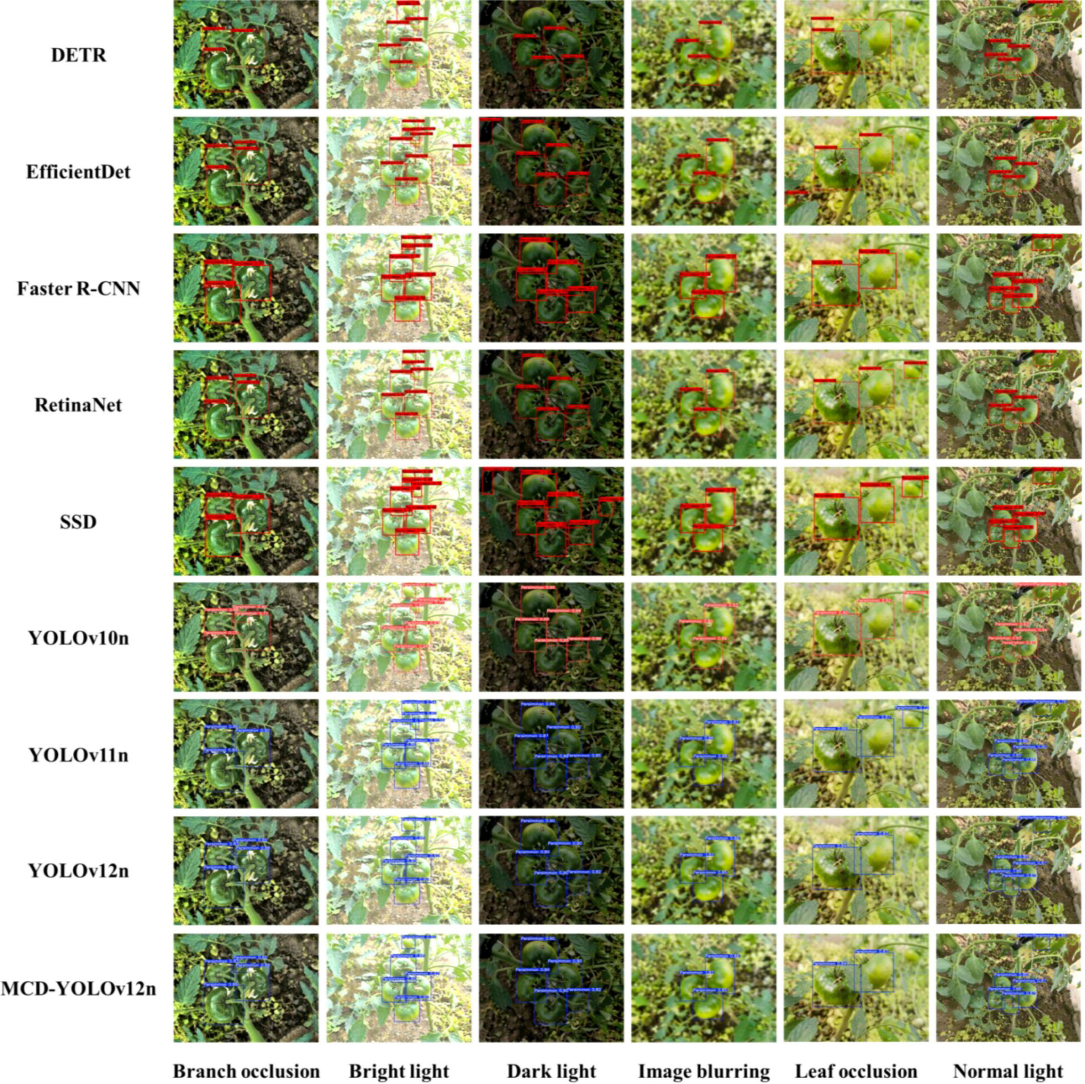
Real-time recognition results of MCD-YOLOv12n compared with classical models under different environmental conditions.

As shown in the results presented in **Figure 11**, substantial differences are observed in the detection stability of different models under complex environmental conditions. DETR can relatively accurately localize fruits under normal light. However, pronounced missed detections occur under branch occlusion and dark light conditions. Its overall mAP is approximately 84.1%, which drops to 76.3% in dark scenes. EfficientDet exhibits clear fruit contour delineation under bright light, but it is prone to producing duplicate detections when fruits are heavily occluded by dense foliage. Faster R-CNN achieves high localization accuracy for large-scale fruits. Nevertheless, its adaptability to small targets is limited under image blurring and dark light conditions, leading to noticeable bounding box offsets. In addition, its overall inference speed is insufficient for real-time applications. Both RetinaNet and SSD maintain high confidence scores under dark light conditions, with confidence levels exceeding 98% for most detected fruits. However, these two models demonstrate poor robustness, as they fail to effectively recognize fruits under normal and bright light, indicating a strong sensitivity to lighting variations. In contrast, YOLO-series models exhibit relatively higher recognition accuracy, with confidence scores exceeding 85% for most fruits across diverse conditions. Among them, YOLOv10 performs the worst, showing lower confidence scores and frequent false detections. YOLOv11 and YOLOv12 achieve superior detection performance among the classical models, although false detections occur under branch occlusion, likely due to the similar color characteristics between branches and immature persimmon fruits. Notably, YOLOv12 and the proposed MCD-YOLOv12n suffer from missed detections under leaf occlusion, whereas YOLOv10 and YOLOv11 do not miss distant immature persimmon fruits. Encouragingly, MCD-YOLOv12n demonstrates the best overall performance among all evaluated models, achieving the highest recognition rate and bounding box confidence. These results further confirm that MCD-YOLOv12n possesses superior practical applicability and strong potential for deployment under complex field conditions.

## 4 Discussion

In this study, an improved model MCD-YOLOv12n, considering both detection accuracy and real-time performance, was constructed to solve the problems of weak saliency, small scale, and complex occlusion of immature persimmon fruit in complex field environments. By incorporating MobileViTv3, CBAM, and DySample into the YOLOv12n framework, the proposed model systematically enhances feature representation of critical fruit characteristics and strengthens multi-scale perception capability. Experimental results obtained under diverse environmental conditions demonstrate that the proposed model exhibits more stable and robust detection performance in the presence of complex illumination variations and occlusions. These findings provide reliable technical support for the automatic perception and early-stage precision management of immature persimmon fruits in field environments.

Based on the experimental results, MCD-YOLOv12n demonstrates a markedly superior overall performance in the task of immature persimmon fruit detection compared with both the baseline model and other improved variants. Specifically, it achieves a precision of 96.30% and an mAP of 95.30%, while maintaining stable detection performance across a wide range of complex environmental conditions. These results indicate that the proposed model achieves synergistic optimization in suppressing background interference, enhancing discriminative fruit features, and improving multi-scale target perception. Notably, the performance gains do not arise from a simple accumulation of individual modules, but rather from the coordinated interaction of multiple architectural improvements at the levels of feature extraction, feature fusion, and spatial reconstruction. Specifically, the MobileViTv3 module enables the model to effectively capture the global structural information of immature persimmons under complex foliage backgrounds. This capability is particularly critical for immature fruits whose color and texture cues are weak or indistinct. Meanwhile, the high-response regions of the MCD-YOLOv12n model are more compact and continuous, with clearly defined fruit boundaries. In addition, the model maintains high detection accuracy under challenging conditions such as image blur and low illumination, while effectively avoiding boundary misalignment and missed detections. This robustness is presumably attributable to the DySample module, which replaces fixed interpolation-based upsampling with a dynamic sampling strategy during feature fusion, allowing the feature reconstruction process to adaptively adjust according to local semantic information. However, the MCD-YOLOv12n model exhibits a relatively lower recall value, which may result from a more conservative detection strategy during the prediction stage, causing some targets with severely blurred boundaries or extreme occlusion to remain undetected. This observation indicates that MCD-YOLOv12n places greater emphasis on balancing precision and overall detection stability, a characteristic that is particularly important for practical applications involving immature persimmon detection. Although MCD-YOLOv12n introduces a moderate increase in parameter count and computational cost compared with the original YOLOv12n, it still achieves an inference speed of 72.5FPS, which is well above the practical requirements for real-time detection. This demonstrates that the model strikes a favorable balance between accuracy enhancement and computational efficiency. Overall, the performance advantages of MCD-YOLOv12n stem from its precise modeling of key characteristics of immature persimmon fruits, thereby providing a solid foundation for stable deployment in complex field environments.

By jointly exploiting shape and color features on the surface of immature persimmon fruits, MCD-YOLOv12n successfully achieves robust fruit identification across diverse scenes. Results from the ablation experiments demonstrate that the proposed model attains a precision of 96.30%, a recall of 89.90%, and an inference speed of 72.5FPS, indicating strong detection performance and real-time capability. To further enhance fruit recognition performance, a variety of advanced technical solutions have been proposed in recent years. Huang et al. (2025) developed a YOLOv8-based fruit recognition approach in which the detection model is first employed to accurately locate and crop fruit regions, followed by classification of the cropped images using a dedicated classification module. This method achieved a classification accuracy of 92.6% on a dataset containing 27 fruit categories. Gao et al. (2024) addressed the challenges of low efficiency and limited accuracy associated with manual identification of young citrus fruits during the seedling stage by proposing a detection model termed YCCB-YOLO. Experimental results demonstrated that the proposed model achieved an accuracy of 91.79% on a seedling-stage citrus fruit dataset, indicating its effectiveness and reliability. These findings suggest that YCCB-YOLO provides an efficient solution for fruit detection in complex orchard environments. Gai et al. (2024) introduced multiplexed coordinated attention into YOLOv8 and replaced the C2f module with an OREPA (online convolutional re-parameterization) module to construct the TL-YOLOv8 model. Combined with transfer learning-based pretraining to improve generalization, their method achieved an accuracy of 84.6% on a blueberry dataset. In comparison, the proposed MCD-YOLOv12n model achieves an identification accuracy of 96.30% for immature persimmon fruits, outperforming the aforementioned methods by 3.7%, 4.51%, and 11.7%, respectively, which highlights its superior recognition capability. Moreover, MCD-YOLOv12n exhibits a significant advantage in detection speed, reaching 72.5FPS, thereby demonstrating its strong potential for real-time fruit recognition applications in complex agricultural scenarios.

This study addresses the challenges of weak visual saliency, small target size, and strong background interference encountered in the detection of immature persimmon fruits under complex field conditions. To this end, an immature persimmon fruit recognition method based on the YOLOv12 framework is proposed, which achieves satisfactory performance in immature persimmon detection tasks. The results provide feasible technical support for early-stage fine-grained field management and decision-making in intelligent agricultural equipment. Nevertheless, image data are highly influenced by natural environmental conditions. Uncontrollable factors such as weather variations, fluctuations in illumination intensity, and rainfall can significantly degrade image quality, thereby imposing higher demands on the model’s generalization capability in extreme scenarios. In addition, differences in fruit morphology and color among persimmon cultivars cannot be fully mitigated when image data are collected from a single region. Accordingly, future research can be further extended and deepened in multiple directions. On the one hand, joint data acquisition across regions, cultivars, and growth stages can be conducted to construct a more diverse and representative dataset of immature persimmon fruits. On the other hand, the proposed model can be further integrated with UAV platforms, intelligent agricultural robots, or multi-sensor systems. Such deep integration would enable a transition from single visual recognition to multi-source collaborative perception. This advancement would provide a more comprehensive and reliable technical framework for intelligent field monitoring and precision regulation.

## 5 Conclusion

This study proposes a real-time recognition method for immature persimmon fruits based on deep learning within the YOLOv12n framework. By integrating MobileViTv3, CBAM, and Dysample modules, the proposed approach effectively enhances the model’s capability to discriminate weak-feature targets and improves its robustness under complex environmental conditions. Based on the experimental results, the following conclusions can be drawn:

1. An image dataset of immature persimmon fruits was constructed to cover complex field environments, including diverse illumination conditions, varying degrees of occlusion, and multiple acquisition angles. In addition, multiple data augmentation strategies were employed, enabling the dataset to exhibit strong generalization performance in challenging field scenarios.
2. The proposed MCD-YOLOv12n model achieved favorable recognition performance on the persimmon fruit image dataset, with precision, recall, and mAP reaching 96.30%, 89.90%, and 95.30%, respectively, while maintaining a detection speed of 72.5FPS. These results effectively demonstrate the model’s effectiveness and practical application potential for immature persimmon fruit recognition tasks.
3. Grad-CAM visualization results indicate that MCD-YOLOv12n focuses more accurately on the main fruit regions during the decision-making process, suggesting that the model possesses strong feature learning capability even under highly variable environmental conditions.
4. Comparative experiments with multiple classical object detection models show that MCD-YOLOv12n exhibits clear advantages across key evaluation metrics, enabling high-precision and low-latency real-time recognition in complex field environments and meeting the practical requirements of agricultural production.

The proposed MCD-YOLOv12n model provides an effective solution for real-time recognition of immature persimmon fruits. It offers valuable technical support for early-stage precision management in orchards and contributes to the advancement of intelligent and smart agriculture.

## Data Availability

The original contributions presented in the study are included in the article/supplementary material. Further inquiries can be directed to the corresponding author.
